# Ultrasonic Processing of Graphene Nanoplatelet–Silver Nanoparticle Composite Coatings for Enhanced Mechanical and Antiviral Properties in Medical Textiles

**DOI:** 10.1021/acsomega.5c06675

**Published:** 2026-05-06

**Authors:** Robert L. F. Liang, Monika Snowdon, Azar Fattahi, Cameron Dean, Derek Eppinghoven, Irfani Ausri, Yun Wu, Steven Phang, Aastha Gandhi, Julie Dang, Tahbit Chowdhury, Shirley Tang, Y. Norman Zhou, Marina Freire-Gormaly

**Affiliations:** † Department of Mechanical Engineering, 7991 York University, 4700 Keele St., Toronto, Ontario M3J 1P3, Canada; ‡ Centre for Advanced Materials Joining, 8430 University of Waterloo, 200 University Ave. West, Waterloo, Ontario N2L 3G1, Canada; § Waterloo Institute of Nanotechnology, University of Waterloo, 200 University Ave. West, Waterloo, Ontario N2L 3G1, Canada; ∥ Feedband Laboratories, 192 Spadina Ave., Toronto, Ontario M5T 2C2, Canada

## Abstract

Respiratory protective textiles increasingly require multifunctional surface treatments that enhance durability and provide active antiviral performance without compromising filtration. In this context, this study develops graphene nanoplatelet–silver nanoparticle (GNP–Ag) and super activated carbon–silver nanoparticle (SAC–Ag) composite coatings for PPE-grade nonwoven fabrics using a room-temperature ultrasonic atomization process. The coatings enhanced mechanical integrity while maintaining baseline filtration efficiency. GNP–Ag increased spunbond grab strength by 54% and, when combined with polyurethane and UV cross-linking, improved meltblown strength by 47%. Surface wettability shifted from hydrophilic to hydrophobic, with meltblown contact angle increasing from 88° to 121°, and wash fastness improved following heat and UV curing. Cytotoxicity assays showed a dose-dependent response, with GNP–Ag exhibiting lower cytotoxicity compared with SAC–Ag. Antiviral testing against human coronavirus OC43 demonstrated a 1.93-log reduction (98.8%) after 10 min for unwashed GNP–Ag fabrics and a 1.43-log reduction (96.3%) after washing. Filtration performance remained unchanged for GNP–Ag and slightly increased for SAC–Ag. These results demonstrate a stable, mechanically reinforcing, and antiviral coating that enhances the protective function of PPE-grade nonwoven fabrics.

## Graphical abstract

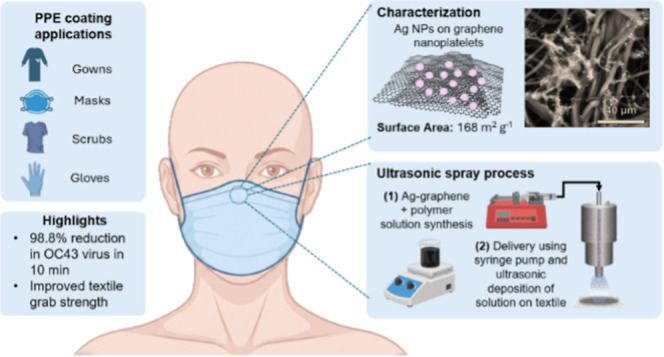

## 1 Introduction

Healthcare workers face significant risks of exposure to pathogens and acquiring infections while treating patients with infectious diseases. The global healthcare landscape has been profoundly impacted by recent outbreaks of diseases such as severe acute respiratory syndrome (SARS), swine flu, Middle East respiratory syndrome (MERS), and the COVID-19 pandemic, necessitating enhanced protective measures, including social distancing, increased use of personal protective equipment (PPE), and lockdown protocols.^1–4^ The COVID-19 pandemic, in particular, has highlighted the critical importance of adequate PPE for frontline healthcare workers. However, recent surveys have revealed concerns about shortages, with 73% of National Health Service (NHS) nurses reporting insufficient PPE and FFP3 respirators and 63% lacking fluid-repellent face masks.^2^

Medical textiles are crucial in healthcare settings, serving multiple functions, including adsorbing bodily fluids, providing barrier protection, and promoting wound healing. These textiles are used in a wide range of products, including PPE (masks, gowns, scrubs, full-body suits, footwear covers, gloves, and drapes), wound dressings, bandages, and bedding. However, these products can also serve as potential sources of the spread of infectious diseases.^5–8^ Although PPE is typically worn for short durations, various viruses, including Severe acute respiratory syndrome coronavirus 2 (SARS-CoV-2),^9^ influenza,^10^ and others,^11–13^ can survive on surfaces for hours, leading to the risk of viral transmission through surface-to-hand and hand-to-hand contact. This underscores the need for enhanced PPE with antipathogenic finishes to reduce the likelihood of microbial contamination on their surfaces.

The increasing prevalence of hospital-acquired infections and the growing risk of pandemics due to globalization have heightened the demand for improved PPE. Healthcare workers face significant risks of exposure to bloodborne pathogens such as Human immunodeficiency virus (HIV) and hepatitis B virus (HBV), necessitating the development of more effective protective gear, such as enhanced surgical gowns.^14^ To address these challenges, researchers are exploring nanotechnology in medical textiles. While there are some restrictions on the use of nanomaterials in specific medical applications, nanoparticles have already been successfully incorporated into various medical products. For instance, silver nanoparticles suspended in polymeric hydrogels have been incorporated into wound dressings to treat skin conditions, demonstrating the potential of nanomaterials to enhance the protective and therapeutic properties of medical textiles.^15–18^ As the healthcare landscape continues to evolve, developing and adopting PPE with antipathogenic coatings may become increasingly necessary for healthcare workers to reduce the transmission of viruses and other pathogens in medical settings.^14,19,20^

Developing washable and reusable functional textiles with antipathogenic properties presents significant challenges. A primary concern is the poor wash fastness of water-soluble antibacterial nanoparticles and surfactants, which compromises the long-term efficacy of treated fabrics.^21–25^ This issue is compounded by the potential for bound antimicrobials to undergo abrasion from textile fibres or deactivation during use, reducing effectiveness over time.^26,27^ The resultant sloughing of nanoparticles raises environmental and health concerns, including the potential for leaching into aquatic ecosystems, subsequent bioaccumulation in food chains, and extended human toxicity through prolonged exposure.^28–30^ While nanotechnology-based treatments offer promising solutions for reducing pathogen burden at lower loadings than traditional methods,^31–35^ they also pose potential cytotoxicity risks that require careful consideration.

This study presents a novel antipathogenic coating with enhanced mechanical integrity and adsorptive properties, advancing protective textiles and materials engineering. The coating formulation incorporates graphene nanoplatelets (GNPs) or super activated carbon (SAC) combined with silver nanoparticles (Ag NPs), leveraging the synergistic effects of these nanomaterials to achieve superior antimicrobial efficacy and mechanical strength. The merits of this type of coating include green chemistry practices, room-temperature coating processes, and ultrasonic processing and UV curing. Furthermore, material and mechanical properties, filtration efficiency, cytotoxicity, and antiviral efficacy were tested against human coronavirus OC43, a surrogate for SARS-CoV-2, as they are both beta-coronaviruses.^36^

## 2 Results and Discussion

### 2.1 Coating Formulation Characterization

Two types of composite dispersions were prepared: graphene nanoplatelet-silver and superactivated carbon and formulation properties given in Table 1. The specific areas of the graphene nanoplatelet and superactivated carbon are 530 m2 g^–1^ and 1800 m2 g^–1^, respectively. The density of the adsorbent is 0.02–0.04 g cc^–1^ and 0.38–0.40 g cc^–1^, respectively. The silver nanoparticles were determined to be 40–60 nm using UV–vis (Figure 1a) and ESEM.

**Table 1:** Summary of Coating Properties

properties	GNP–Ag	SAC–Ag
specific surface area	168 m^2^ g^–1^	1521 m^2^ g^–1^
pore volume (cm^3^ g^–1^)	0.333	0.742
density of adsorbent	0.02–0.04 g cc^–1^	0.38–0.40 g cc^–1^
thickness of adsorbent particulate	1.5 nm (GNP)	N/A
antiviral agent particle size	40–60 nm	40–60 nm
zeta potential	10 mV	14 mV
pH	5.51	4.69
dispersion stability	10 days (qualitative)	10 days (qualitative)

**Figure 1:**
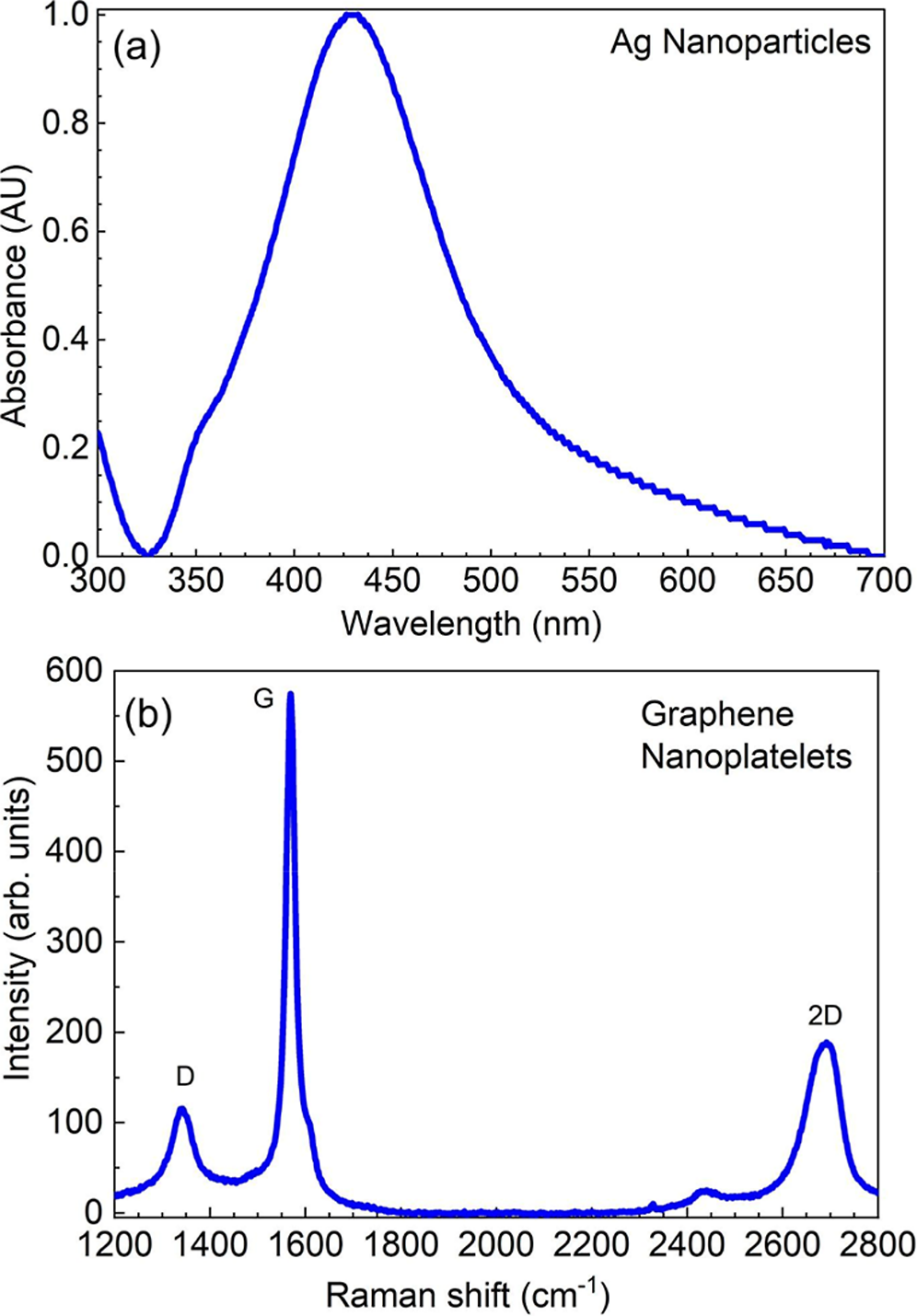
(a) Raman spectra of graphene nanoplatelets (532 nm, 10 mW output) and (b) UV–visible spectra of synthesized Ag solution.

The Raman spectroscopy results shown in Figure 1b provide structural characteristics of the GNP used in the coating formulation. The Raman spectra reveal three prominent peaks characteristic of graphene-based materials: the D band (∼1350 cm^–1^), G band (∼1580 cm^–1^), and 2D band (∼2700 cm^–1^). The intensity ratio of the D band to the G band (*I*_D_/*I*_G_) is a crucial parameter for assessing the degree of defects or disorder in the graphene structure. In this case, the *I*_D_/*I*_G_ ratio of 0.27 indicates a relatively low defect level. This low defect density is consistent with the high surface area of the nanoplatelets, as fewer defects generally correlate with larger, more intact graphene sheets.^37^

The intensity ratio of the 2D band to the G band (*I*_2D_/*I*_G_) provides information about the number of graphene layers present.^38^ The intensity ratio of the observed *I*_2D_/*I*_G_ ratio of 0.33 is significantly lower than what would be expected for single-layer graphene (typically >1). This low ratio indicates multiple graphene layers stacked together, characteristic of graphene nanoplatelets or exfoliated graphite rather than single-layer graphene. Furthermore, the shape and full width at half-maximum (fwhm) of the 2D band also corroborate the multilayer nature of the sample. In multilayer graphene or graphite, the 2D band tends to be broader and may show asymmetry or splitting compared to the sharp, symmetric peak observed in single-layer graphene.^38^

The dispersibility of the coatings was measured using a sedimentation technique and a time-lapse camera. The sedimentation revealed that the samples remained stable after 10 days, with no noticeable visual change in the sediment (Figure S1).

### 2.2 Fabric Testing

#### 2.2.1 Assessment of Surface Characteristics

Assessing surface characteristics through ESEM imaging provides crucial insights into the structure and composition of the GNP–Ag coated fabric used in N95-grade filtration discs (spunbond - meltblown - spunbond fabric). The images show a network of fibres interspersed with graphene nanoplatelets (GNPs) and silver nanoparticles (Ag NPs). Figure 2a–d depicts the GNP–Ag coated spun bond layer under backscattering electron mode, highlighting the distribution of both GNPs and Ag NPs throughout the fabric matrix. The GNPs appear as thin, sheet-like structures dispersed among the fibres, while the Ag NPs are visible as bright, discrete particles. Notably, a higher concentration of Ag NPs within the GNP layers suggests a potential synergistic arrangement that could enhance the material’s antimicrobial properties. The energy dispersive X-ray spectroscopy (EDS) measurements (Figure 2e) confirm that bright particles correspond to silver, validating the presence and distribution of Ag NPs within the fabric structure. The elemental composition analysis provided in the image further corroborates the presence of key elements in the coated fabric. The table shows a predominance of carbon (91.51 atomic %), as expected given the fabric’s organic nature and the presence of GNPs. Oxygen (7.07 atomic %) is also present, likely from the fabric material and possible surface oxidation. The detection of Ag (1.16 at %) confirms the successful incorporation of Ag NPs into the fabric structure.

**Figure 2:**
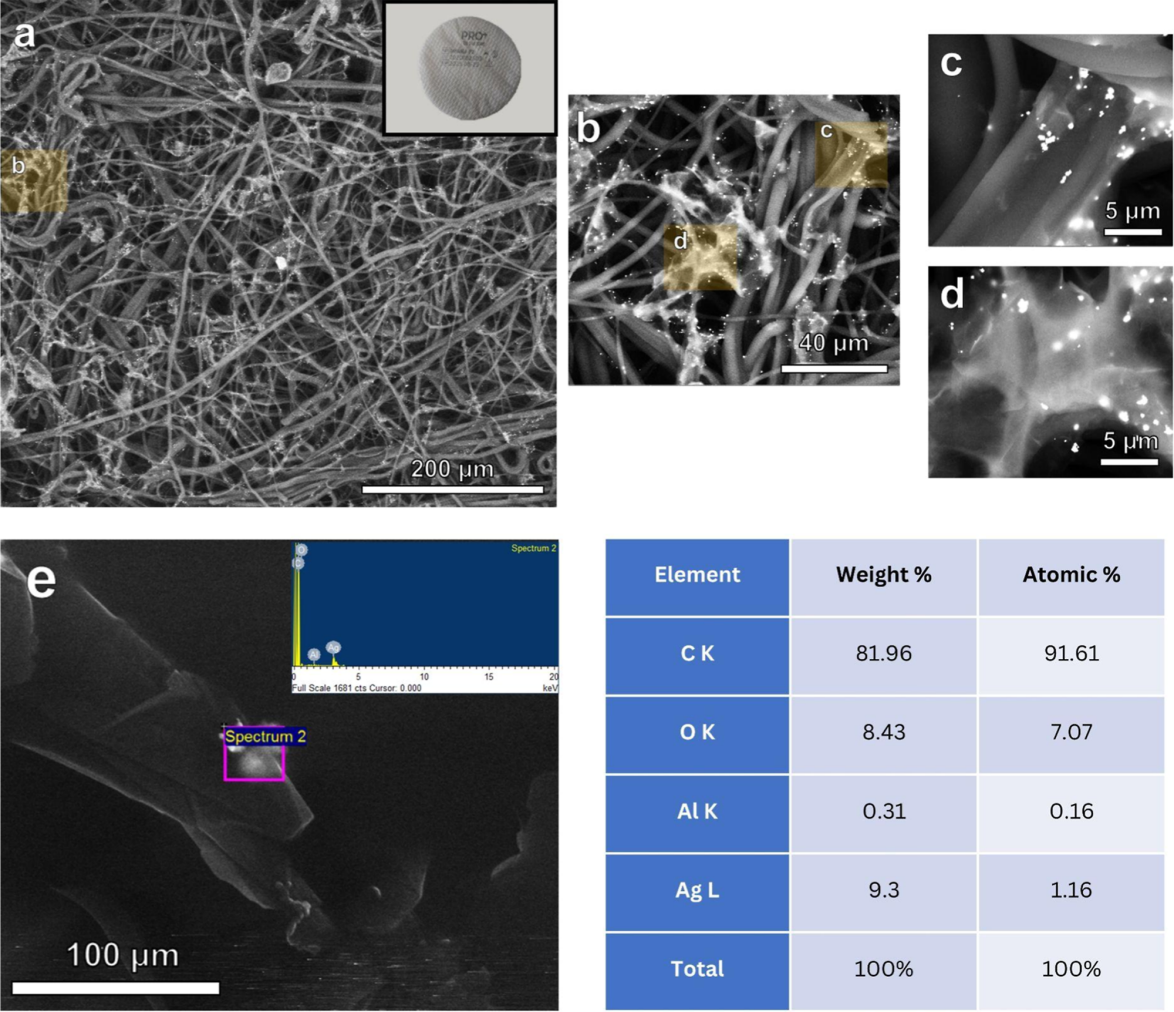
(a–d) ESEM images of GNP–Ag coated 3-ply filter discs containing silver nanoparticles (Ag NP) and graphene nanoplatelet (GNP) at various magnifications and (e) energy dispersive spectroscopy of GNP–Ag coated 3-ply fabric discs.

Figure 3a–c provides a detailed view of SAC–Ag coated 3-ply filter discs under backscattering electron mode. The images reveal a network of interwoven fibers characteristic of the 3-ply filter discs, with the SAC and Ag NPs dispersed throughout the fabric matrix. At higher magnifications (Figure 3b,c), the SAC particles appear to be darker, irregularly shaped structures. At the same time, the Ag NPs are visible as bright, discrete particles, consistent with their higher atomic number and consequent stronger electron backscattering. The distribution of these components is relatively uniform across the observed area.

**Figure 3:**
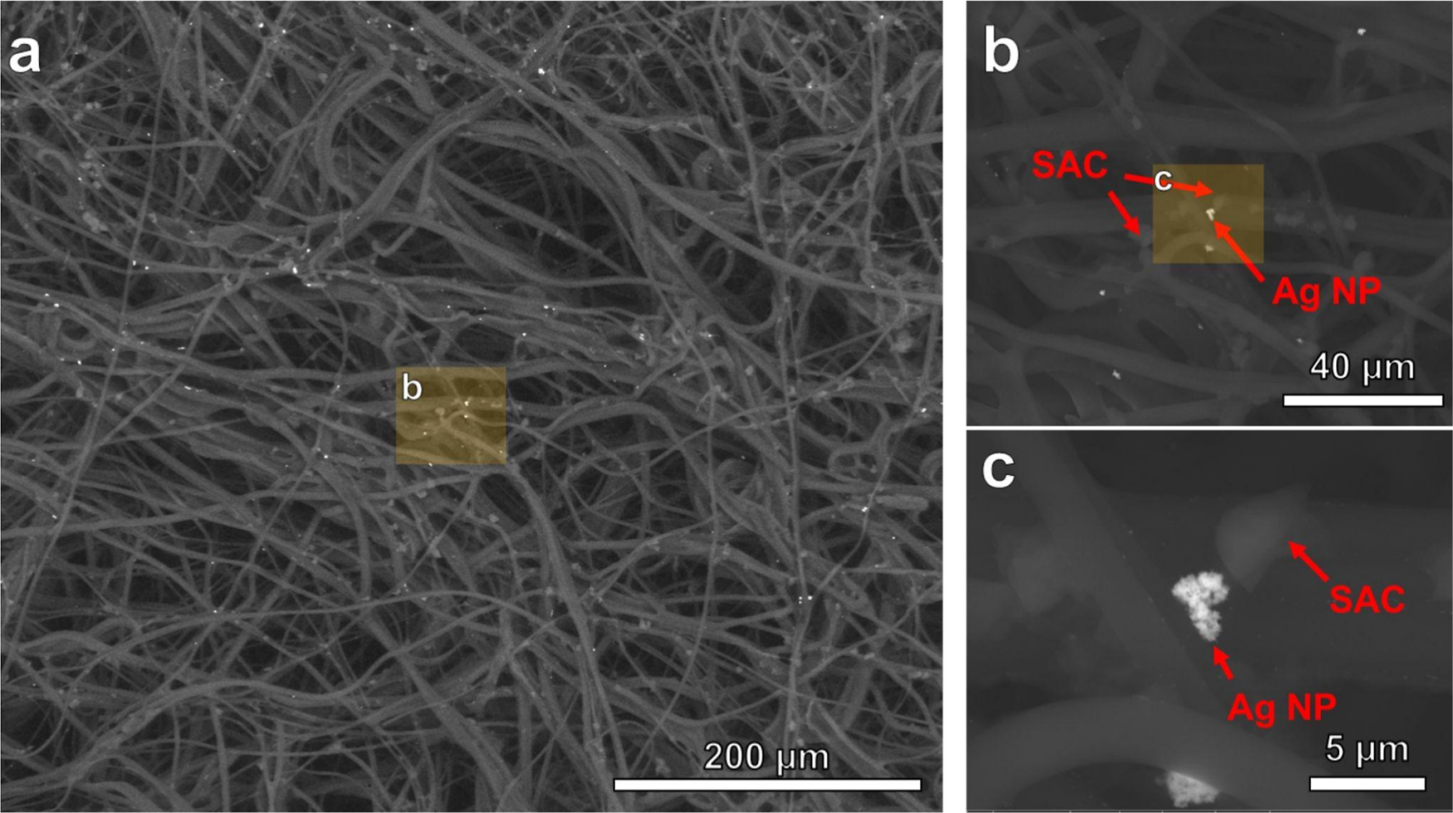
(a–c): ESEM images of coated 3-ply filter discs containing silver nanoparticles (Ag NP) and super activated carbon (SAC).

Figure 4 presents Raman spectroscopy, XRD, and FTIR-ATR analyses that confirm the successful integration of the GNP–Ag coating onto polypropylene spunbond fabrics and verify that the coating does not alter the underlying polymer structure. In Figure 4a, the Raman spectra of coated spunbond fabrics show reduced intensity across the 1300–1500 cm^–1^ region due to partial attenuation from the discontinuous distribution of graphene sheets on the polypropylene fabric surface.

**Figure 4:**
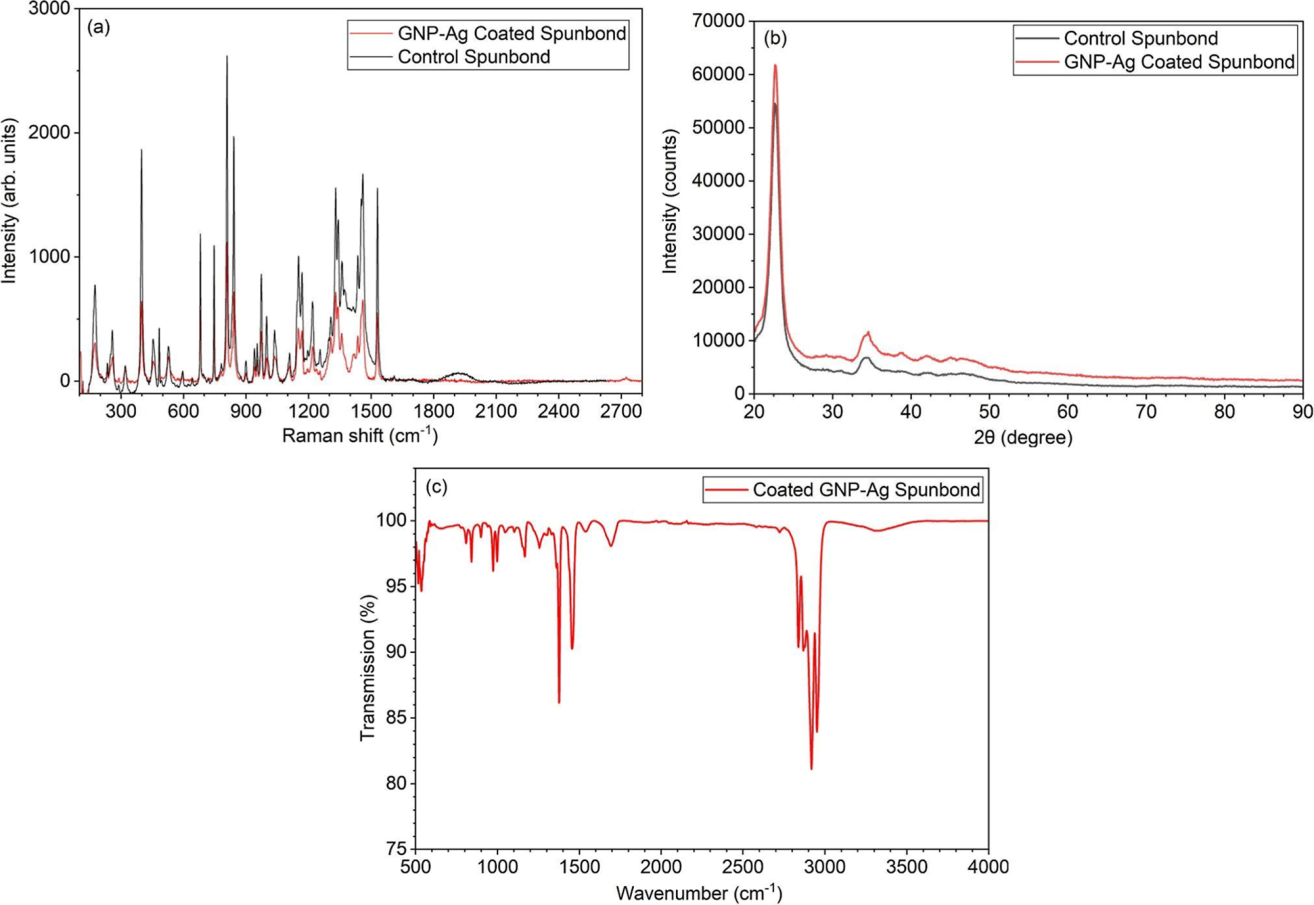
(a) Raman spectroscopy, (b) XRD, and (c) FTIR-ATR analyses of GNP–Ag-coated polypropylene spunbond fabrics as part of 3-ply filter discs.

Figure 4b shows the XRD patterns of coated and control spunbond fabrics. Both samples retain the dominant polypropylene diffraction peak at ∼21°, demonstrating that the ultrasonic atomization, drying, and UV processing steps do not disrupt polymer crystallinity. A slight increase in background intensity in the coated fabric is consistent with the presence of graphene nanoplatelets and nanosilver dispersed on the surface. Importantly, no new crystalline phases are observed, supporting that the coating forms a surface-level composite layer without altering bulk fabric structure.

Figure 4c presents the FTIR-ATR spectrum of the coated spunbond fabric, which shows the standard polypropylene absorption bands: CH_2_ stretching near 2950–2840 cm^–1^, CH bending at ∼1450–1375 cm^–1^, and skeletal vibrations below 1300 cm^–1^. The absence of new peaks confirms that neither the GNP–Ag coating nor the UV curing induces chemical modification of the polypropylene. The coating signal is not dominant in FTIR due to the low mass fraction of the applied nanomaterials and the strong infrared absorption of the polymer.

#### 2.2.2 Mechanical Integrity Testing

For spunbond fabrics, the coating process resulted in a 54% enhancement in grab strength compared to virgin control samples and a 25% improvement over heat-treated controls, as shown in Figure 5a. This increase in tensile strength suggests that the GNP–Ag coating effectively reinforces the fiber network, potentially through the formation of interfiber bonds or by filling voids within the fabric structure. In contrast, the meltblown fabrics responded differently to the coating application (Figure 5b). While no significant improvement in grab strength was observed between the heated control and the coated meltblown samples, the heat treatment process had a detrimental effect, reducing grab strength by 20–30% compared to the virgin meltblown fabric. This reduction in strength due to heat treatment demonstrates the thermal sensitivity of meltblown structures. Applying the GNP–Ag coating did not significantly alter the elongation at break for spunbond or meltblown fabrics compared to their respective virgin and heat-treated controls. This preservation of extensibility demonstrates that the coating enhances strength without compromising the fabric’s ability to deform under stress, a crucial factor for maintaining comfort and flexibility in protective textiles.

**Figure 5:**
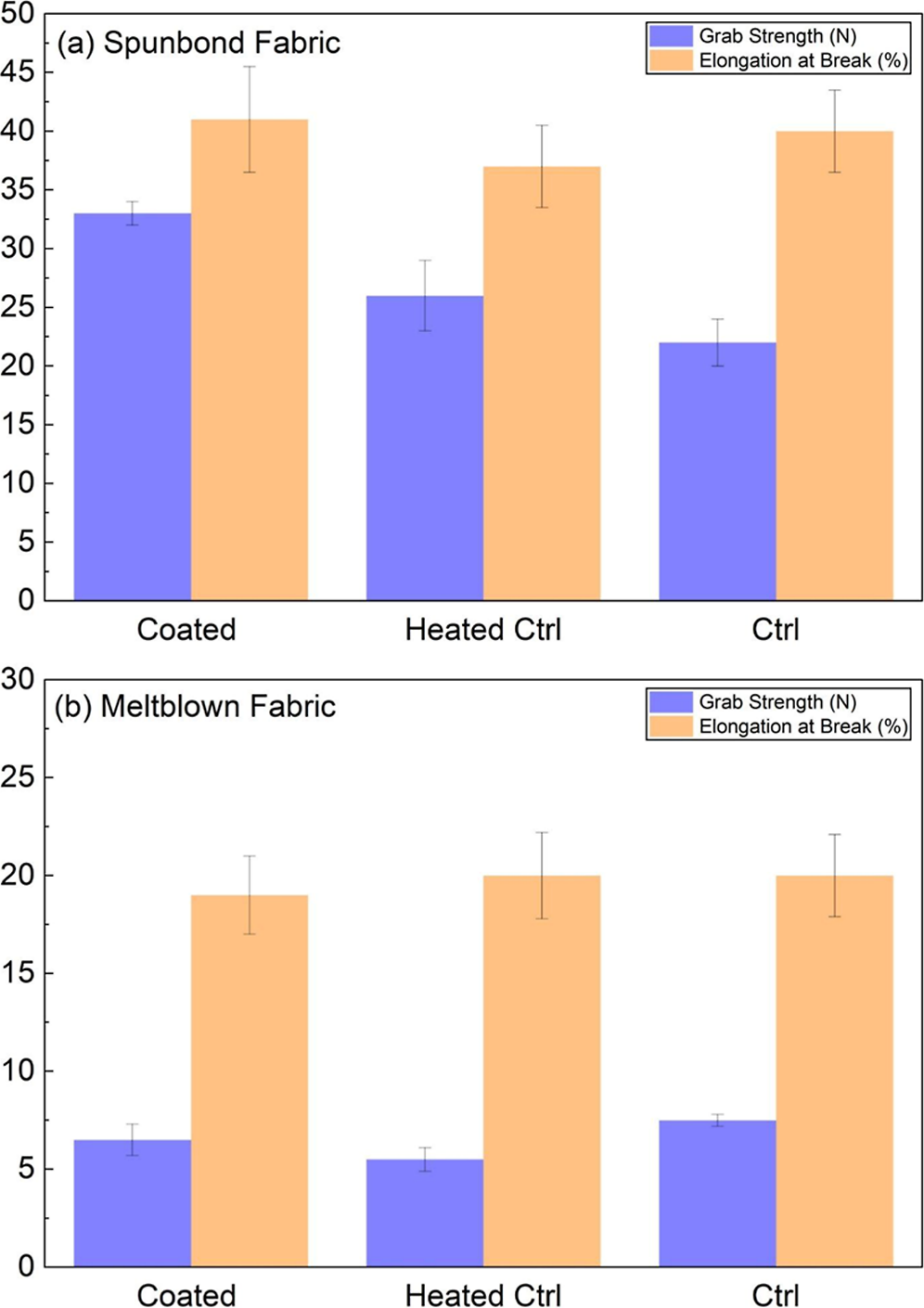
Mechanical properties of spunbond and meltblown fabrics with and without coating treatment. (a) Spunbond fabric: grab strength (N) and elongation at break (%) for GNP–Ag coated filter, heated control, and untreated control samples. (b) Meltblown fabric: grab strength (N) and elongation at break (%) for GNP–Ag coated filter, heated control, and untreated control samples.

#### 2.2.3 Effect of Polyurethane Coating Additive and UV Treatment on Meltblown Fabric

Polyurethane diol (PUD), a tackifier, and benzophenone, a UV initiator, were added into the coating mixtures to improve the grab strength of meltblown fabric. Benzophenone can initiate cross-linking reactions with polyurethane diol and PVP under UV irradiation at wavelengths below 350 nm. A UV cross-linking agent was used to achieve faster reaction times during manufacturing operations, and autocrosslink was not used due to residual heat in the process. The UV treatment has also been shown to meet the N95 meltblown filtration performance requirement at a UV dose 10 times higher than that used for coatings (see Section S1 in Supporting Information). The study in Section S1 of the Supporting Information uses low-pressure mercury lamps that emit higher-energy radiation (λ = 254 nm) than the UV-LEDs (λ = 280 nm) used to cross-link the coated fabrics.

The experimental procedure involved evaluating grab strength for both uncoated and coated samples after heat treatment at 80 °C for 30 min. A one-way ANOVA was performed to assess the significance of the results, as illustrated in Figure 6. All UV-treated coated samples exhibited significantly improved grab strength compared to the control sample at the α = 0.05 significance level. Adding the PUD additive alone, without UV treatment, also resulted in a significant increase in strength over the control. However, GNP–Ag and SAC–Ag coatings without the tackifier or UV treatment did not show a significant improvement in grab strength compared to the control. The combination of GNP–Ag coating with both a PUD additive and UV treatment demonstrated the greatest improvement in grab strength, surpassing both the control and SAC–Ag samples.

**Figure 6:**
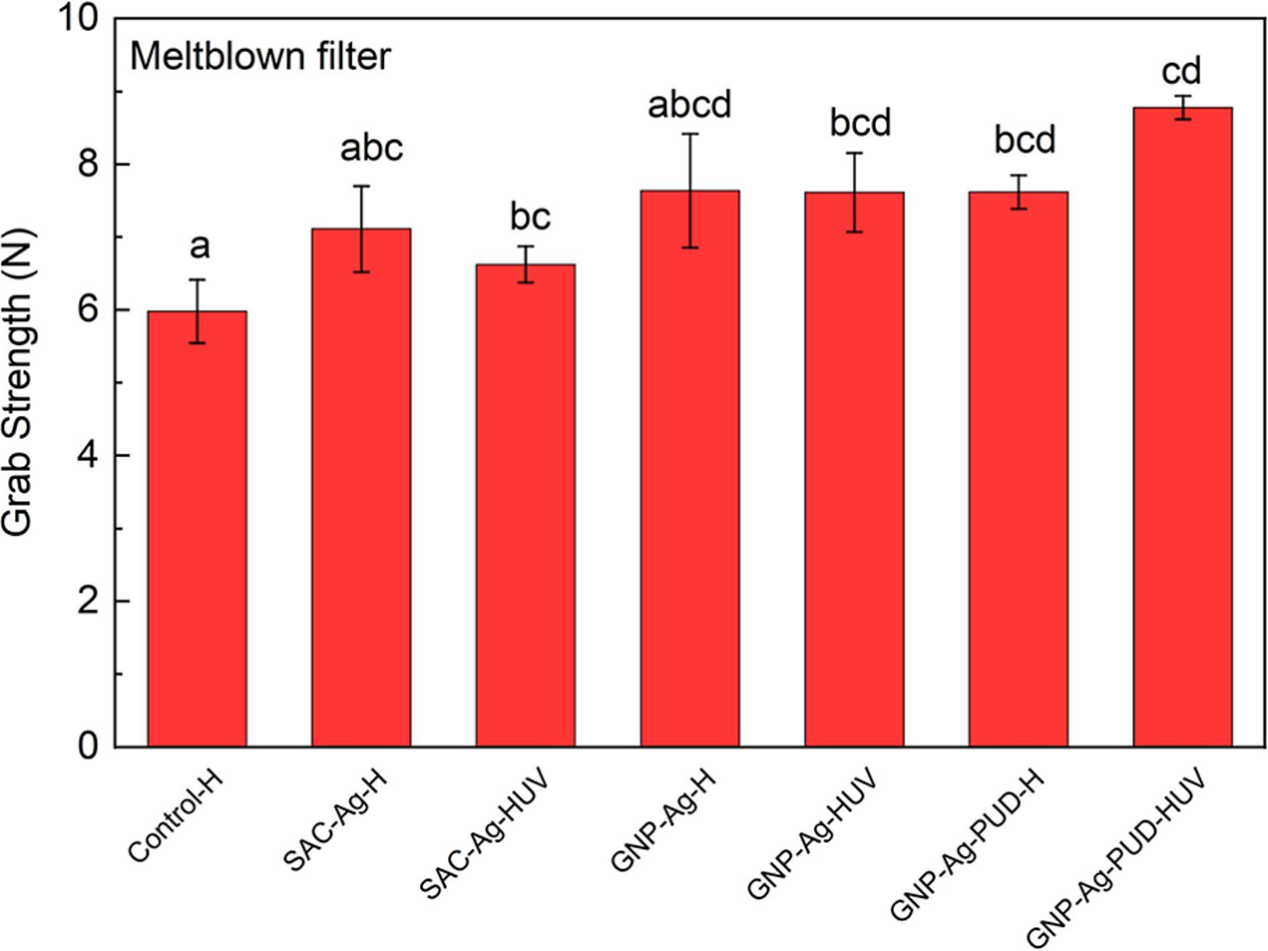
Grab strength of coated meltblown filters using three types of coatings (SAC–Ag, GNP–Ag, and GNP–Ag-PUD) and two treatments (H—heat and HUV—heat and UV). The significance was evaluated at α = 0.05 using the Tukey means comparison method.

The most pronounced results were obtained with UV cross-linking treatment of GNP–Ag-PUD-coated meltblown filters. This treatment led to a 47% improvement in grab strength compared to uncoated meltblown filters, a 33% improvement over cross-linked SAC–Ag meltblown filters, and a 15% improvement over cross-linked GNP–Ag meltblown filters without PUD. These findings highlight the synergistic effect of combining GNP–Ag coating, PUD additive, and UV treatment in significantly enhancing the mechanical properties of meltblown fabrics.

#### 2.2.4 Contact Angle

Contact angle measurements were conducted to determine a fabric’s degree of hydrophilicity or hydrophobicity (Table 2). The control sample exhibited a contact angle of 88° for the polypropylene MB fabric, indicating a slightly hydrophilic surface. However, after GNP–Ag treatment, the contact angle increased dramatically to 121°, representing a 37.5% increase. This substantial change transformed the MB fabric surface from hydrophilic to strongly hydrophobic, as evidenced by contact angles above 90°. This significant increase in hydrophobicity could potentially enhance the fabric’s water repellency and affect its filtration properties.

**Table 2:** Contact Angles of Coated and Uncoated Filtration Media Substrates

type of substrate	contact angle (^o^)		
	control	GNP–Ag treatment	SAC–Ag treatment
polypropylene meltblown	88	121	120
polypropylene spunbond	118	115	116

In contrast, the polypropylene SB fabric showed different behavior. The control SB fabric had a contact angle of 118°, indicating a hydrophobic surface. After GNP–Ag treatment, the contact angle decreased slightly to 115°. This minor change suggests that the GNP–Ag treatment had minimal impact on the surface properties of the SB fabric, maintaining its hydrophobic nature. SAC–Ag treatment should have properties similar to those of GNP–Ag treatment.

The dramatic increase in hydrophobicity for the MB fabric could be particularly beneficial for applications requiring water repellency or enhanced droplet capture. In contrast, the maintained hydrophobicity of the SB fabric suggests that its original surface properties were preserved mainly after treatment.

#### 2.2.5 Wash Testing

The wash testing results in Table 3 provide insight into the coating integration and stability of GNP–Ag-treated polypropylene fabrics under liquid conditions. The test was conducted on both SB and MB polypropylene fabrics, comparing untreated control samples with GNP–Ag treated samples subjected to different processing steps: air drying, heat curing, and combining heat and UV curing. The control sample showed a low turbidity of 2.76 ± 0.45 NTU for the polypropylene SB fabric. The air-dried GNP–Ag treated sample exhibited the highest turbidity at 16.85 ± 1.83 NTU, indicating significant particle release. However, heat curing and heat + UV curing substantially reduced turbidity to 7.57 ± 0.45 NTU and 7.14 ± 0.95 NTU, respectively, suggesting improved coating adhesion. The polypropylene MB fabric control sample had a turbidity of 2.48 ± 0.43 NTU. Heat curing resulted in the highest turbidity, 16.60 ± 1.05 NTU, while heat + UV curing reduced it to 11.75 ± 1.98 NTU. This indicates that for MB fabric, the combination of heat and UV treatment was more effective in improving coating adhesion than heat alone.

**Table 3:** Wash Testing for Untreated and GNP–Ag Treated Samples

processing step	polypropylene spunbond fabric		polypropylene meltblown fabric	
	turbidity (NTU)	[Ag]^+^ concentration (ppm)	turbidity (NTU)	[Ag]^+^ concentration (ppm)
control	2.76 ± 0.45	<0.1^a^	2.48 ± 0.43	<0.1^a^
air dry	16.85 ± 1.83	<0.1^a^		
heat cure	7.57 ± 0.45	<0.1^a^	16.60 ± 1.05	<0.1^a^
heat + UV cure	7.14 ± 0.95	<0.1^a^	11.75 ± 1.98	<0.1^a^

a Lower detection limit (LDL).

Notably, the [Ag]+ concentration remained below the lower detection limit (<0.1 ppm) for all samples and processing steps, suggesting minimal silver ion release into the wash water. These results demonstrate that heat treatment, especially when combined with UV curing, enhances the adhesion of the GNP–Ag coating to both SB and MB polypropylene fabrics. This improved adhesion is evidenced by the reduced turbidity in the wash water compared to air-dried samples, indicating fewer particles being released during vigorous washing.

### 2.3 Cytotoxicity Testing

The cytotoxicity testing protocol was designed to evaluate the effects of the antipathogenic coatings on cellular viability. Control and antiviral-treated specimens were first sterilized using UV irradiation and subsequently vacuum-sealed to maintain sterility. To simulate exposure conditions, the specimens were immersed in 20 mL of washing solution, specifically HeLa cell medium, and subjected to a rigorous vortexing procedure consisting of five 5 s intervals. This process aimed to extract leachable components from the coated fabrics that might impact cell health.

Cell viability was assessed using the Alamar Blue assay, a well-established method for quantifying metabolic activity in cell populations. This fluorometric/colorimetric growth indicator relies on metabolically active cells reducing resazurin to resorufin, resulting in a measurable color change. The assay provides a sensitive and reliable means of evaluating cytotoxicity across various concentrations and exposure times.^39–41^

For the 15 min contact time experiments, cell viability was measured across a series of dilutions of the washing solution, ranging from 10^0^ to 10^–5^. The results, as depicted in Figure 7, reveal distinct patterns of cytotoxicity for different coating formulations. The control specimens maintained high cell viability (>95%) across all dilutions, serving as a baseline for comparison. In contrast, the GNP–Ag coated specimens exhibited a dose-dependent cytotoxic effect, with cell viability dropping to approximately 75% at the highest concentration (10^–2^ dilution) before recovering to control levels at higher dilutions. The SAC–Ag coated specimens showed a more pronounced cytotoxic effect, with cell viability reduced to about 60% at the 10^–2^ dilution. This differential response suggests that the carbon nanomaterial sloughing from the coated fabric is crucial in determining its cytotoxic potential.

**Figure 7:**
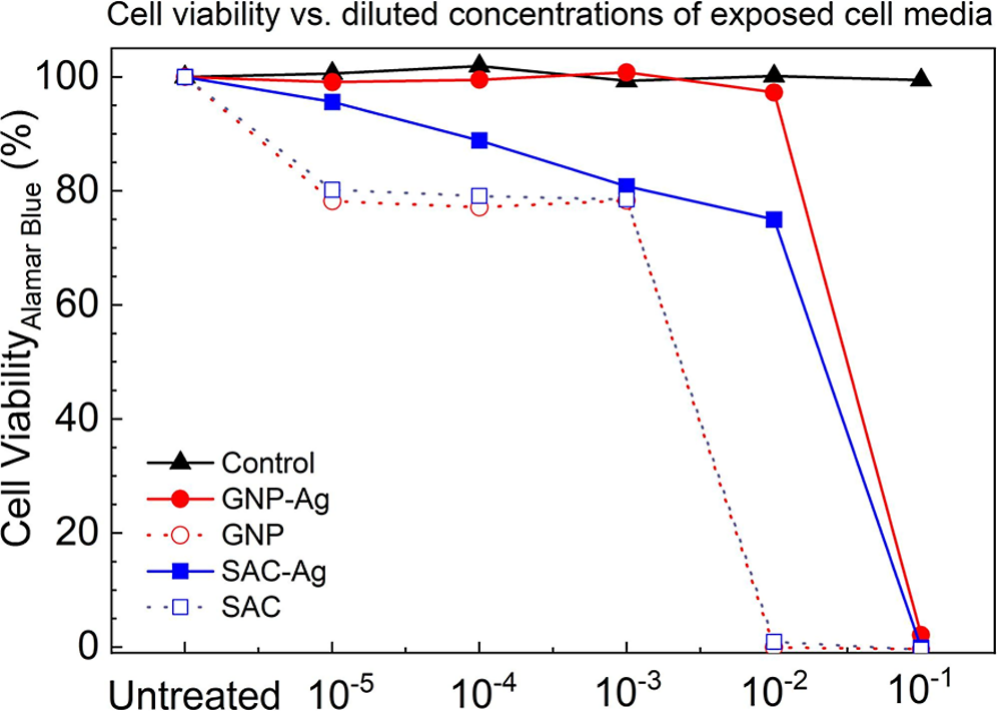
Cell viability as a function of diluted concentrations of cell media wash solution with control, GNP–Ag, GNP, SAC–Ag, and SAC coated fabrics (2 cm × 2 cm) at a 15 min contact time.

### 2.4 Respirability Testing

Respirability testing was conducted to evaluate the potential release of particulate matter from the coated fabrics during simulated breathing conditions. The assessment utilized a TRX DustTrak aerosol monitor to quantify particles’ respirable fraction (PM4). Results revealed that the control mask exhibited an average respirable fraction (PM4) of 0.002 mg m^–3^, while the GNP–Ag and SAC–Ag treated masks demonstrated slightly higher average values of 0.003 mg m^–3^ and 0.007 mg m^–3^, respectively. These findings indicate a marginal increase in respirable particle release from the treated masks compared to the control, with the SAC–Ag treatment showing the highest, albeit still low, level of particle emission.

### 2.5 Filtration Performance

The GNP–Ag and SAC–Ag coated fabrics were used to quantify particle filtration efficiency and pressure drop (Table 4). The average NaCl particle size was 75 nm. The results of Table 4 indicates that the NaCl particle filtration efficiency test using coated and uncoated ASTM 2 3-ply fabric (polypropylene spunbond—polypropylene meltblown—polypropylene spunbond) showed no change or only a slight improvement in filtration efficiency, and the pressure drop was the same across all samples tested. The coated sample GNP–Ag utilized graphene nanoplatelets as the adsorbent material in the multifunctional, antipathogenic treatment, whereas the coated sample SAC–Ag consisted of the composition in GNP–Ag except graphene nanoplatelets, which were substituted with super activated carbon. Sample SAC–Ag demonstrated improved particle filtration efficiency, whereas the GNP–Ag sample demonstrated similar particle filtration efficiency compared to the uncoated samples at room temperature and samples heated at 80 °C for 30 min. The filtration efficiency in SAC–Ag over GNP–Ag is likely due to the higher surface area of the superactivated carbon used in SAC–Ag compared to the graphene nanoplatelet surface area.

**Table 4:** Particle Filtration Efficiency (PFE) and Pressure Drop (Δ*P*) of ASTM 2 Fabric Samples with and without GNP–Ag and SAC–Ag Coatings

sample	PFE	σ	Δ*P*
ASTM 2 fabric (80 °C, 30 min)	75.4%	±5%	4.3 mmH_2_O
ASTM 2 fabric	72.1%	±4%	4.3 mmH_2_O
GNP–Ag on ASTM 2 fabric	74.7%	±7%	4.3 mmH_2_O
SAC–Ag on ASTM 2 fabric	78.0%	±6%	4.3 mmH_2_O

### 2.6 Preliminary TCID50 Testing on GNP–Ag Samples

The 50% tissue culture infected dose (TCID50) method was employed to evaluate the antiviral activity of coated textiles against human coronavirus OC43, a surrogate for SARS-CoV-2.^36^ Before testing, samples were washed to reduce cytotoxicity by vortexing the fabrics in MilliQ water for 5 s, repeated 5 times, to remove excess unbound particulates from the coating process. The initial virus titer was 5 log/mL. Viral stocks were passed through treated and untreated textiles for a 10 min contact time, then serially diluted and inoculated into NIH/3T3 cells.^42^ Antiviral effects were determined after a 10 day culture period using the TCID50 method. Virus-induced cell death was observed by analyzing cell morphology using an inverted light microscope in brightfield white light mode. The antiviral tests showed a 2-log reduction for GNP–Ag samples and a 1.43-log reduction for washed GNP–Ag samples without the antiviral agent. As presented in Table 5, these results demonstrate significant viral titer reductions, with the unwashed GNP–Ag sample achieving a 1.93 log reduction, corresponding to a 98.8% reduction in viral titer after 10 min of contact time.

**Table 5:** Preliminary Virologic Testing

test virus	contact time	sample ID	virus titer (TCID_50_ per carrier)	log_10_ reduction	percent reduction (%)
human coronavirus OC43 (ATCC)	0 min		1.28 × 10^5^		
	10 min	control fabric	6.90 × 10^4^	0.27	46.1
	10 min	GNP–Ag (washed)	4.70 × 10^3^	1.43	96.3
	10 min	GNP–Ag (unwashed)	1.49 × 10^3^	1.93	98.8

This work, which uses GNP–Ag on medical textiles, demonstrated antiviral activity against human coronavirus OC43, achieving a 98.8% reduction in 10 min for unwashed samples and a 96.3% reduction for washed samples. This performance is comparable to some of the best results reported in the literature for other materials and viruses (Table 6). For instance, Cu_2_O polyurethane coatings showed slightly higher efficacy against SARS-CoV-2, with a 99.9% reduction in 1 h on stainless steel.^43^ Ag NP-anchored graphene also exhibited potent inhibition against feline coronavirus at 10–100 mg/mL concentrations.^44^ Cotton textiles with cranberry extracts achieved ∼99% viral inactivation of SARS-CoV-2 in just 1 min, which is faster than our results but on par in terms of reduction percentage.^45^ Ag/Cu-zeolite in plastic showed similar efficacy to our unwashed samples, with 98.6% reduction of human coronavirus 229E after 24 h.^46^ The reactive Ag ink with PDMS on PET medical textile demonstrated 95.2% reduction of Herpes simplex virus after 3 h of ultrasonic washing, which is slightly lower than our washed sample results, but still significant.^47^

**Table 6:** Antiviral Activity of Various Materials in Literature

active component	host cell	virus	reduction (%)	refs
Ag NP/GNP on medical textile	NIH 3T3 cells	human coronavirus OC43	98.8% in 10 min on unwashed GNP–Ag treated textile and 96.3% in 10 min on washed GNP–Ag treated textile	this work
Cu_2_O/polyurethane coatings	Vero E6 cells	SARS-CoV-2	99.9% in 1 h on stainless steel	^43^
Ag NP-anchored graphene oxide	CRFK cells	feline coronavirus (FCoV)	inhibition at 10–100 mg/mL in suspension	^44^
cotton textile with cranberry extracts	Vero E6 cells	SARS-CoV-2	∼99% viral inactivation in 1 min on cotton textile	^45^
Ag/Cu-zeolite in plastic (5 wt %/wt)	MRC-5 cells	human coronavirus 229E	98.6% after 24 h on surface of plastic coupons	^46^
reactive Ag ink with PDMS on PET medical textile	A549 cells	herpes simplex virus (HSV-1)	98.2% after 5 h using ultrasonically washed PET medical textile	^47^
Cu-IT (copper ion-textile)	MDCK cells	influenza A virus (IAV)	high antiviral activity after 3 and 24 h on textile	^71^

The antiviral activity of the GNP–Ag coating arises from the combined effects of Ag and GNP. Ag nanoparticles release Ag^+^ ions that interact with viral envelope proteins, disrupt membrane integrity, and interfere with viral RNA through binding to nucleic acids.^48–50^ Graphene nanoplatelets provide a high-surface-area adsorptive interface that immobilizes virions through van der Waals and electrostatic interactions, reducing their ability to reach host cells. The multilayer graphene structure also presents defect sites and sharp edges that can induce local physical disruption and oxidative stress.^51,52^ These processes act synergistically, resulting in (i) rapid adsorption of virions onto graphene surfaces, (ii) Ag^+^-mediated damage to viral membranes and proteins, and (iii) combined structural and oxidative stress leading to loss of infectivity. This mechanism aligns with previously reported antiviral behavior of Ag-graphene systems and supports the rapid reductions observed in the TCID50 assays.^50,51,53–56^

The results are particularly noteworthy given the relatively short contact time (10 min) compared to some of these studies listed in Table 6. The effectiveness of the GNP–Ag coating, even after washing, suggests a durable antiviral effect that could be valuable in real-world applications where PPE may be exposed to moisture or repeated use. However, further studies with SARS-CoV-2 and other relevant pathogens, as well as investigations into the long-term stability and safety of the coating, are needed to fully assess its potential for real-world applications.

## 3 Conclusion

This work demonstrates that GNP–Ag and SAC–Ag antipathogenic coatings, applied through a green, room-temperature ultrasonic atomization process, substantially enhance the mechanical, antiviral, and surface properties of nonwoven medical textiles while preserving essential filtration performance. The coatings increased spunbond grab strength by 54% and, when combined with polyurethane diol and UV curing, improved meltblown strength by 47% without reducing elongation at break. Surface characterization confirmed uniform nanoparticle distribution, and contact angle measurements showed that GNP–Ag converted meltblown fabrics from hydrophilic to strongly hydrophobic (88°–121°). Wash testing demonstrated improved coating adhesion after heat and UV curing, with turbidity reduced and silver ion release remaining below detection limits. Cytotoxicity assessments showed a dose-dependent response, with GNP–Ag exhibiting lower cytotoxicity than SAC–Ag. Particulate respirability testing indicated only marginal increases in PM4 levels, confirming low particle release. Filtration measurements showed no loss of particle capture efficiency for GNP–Ag and a slight improvement for SAC–Ag. Preliminary antiviral testing against human coronavirus OC43 demonstrated a 1.93-log (98.8%) reduction in 10 min for unwashed GNP–Ag fabrics and 1.43-log (96.3%) for washed fabrics, indicating durable antiviral activity. Together, these results show that the developed coatings offer a stable, mechanically reinforcing, hydrophobic, and antiviral treatment compatible with roll-to-roll UV processing and well suited for enhancing the protective performance of PPE-grade nonwoven fabrics.

## 4 Experimental Section

### 4.1 Reagents and Chemicals

Graphene nanoplatelets (GNP, 97%) were obtained from Cheaptubes (Vermont, United States). Super activated carbon was received from US Nanomaterials from SkySpring Nanomaterials Inc. (Texas, United States). Silver nitrate (AgNO_3_, >99.0%), polyvinylpyrrolidone (PVP K30) ammonium hydroxide (NH_4_OH, 30% NH_3_ basis), ascorbic acid (C_6_H_8_O_6_, 99%), sodium citrate dihydrate (HOC­(COONa)­(CH_2_COONa)_2_·2H2O, >99%) isopropanol ((CH_3_)_2_CHOH, 99.9%), cetyltrimethylammonium bromide (CTAB, CH_3_(CH_2_)_15_N­(Br)­(CH_3_)_3_, >98%), polyurethane diol (PUD, average Mn ∼320, 88 wt. % in H2O), and benzophenone ((C_6_H_5_)_2_CO, >99%) were obtained from Sigma Aldrich. Solvents and chemicals for synthesis were obtained from Sigma Aldrich at >99% purity. Ultrapure water (18.2 mΩ cm resistivity at 25 °C) was obtained from a MilliQ Integral Water Purification System by EMD Millipore.

### 4.2 Synthesis of Silver Nanoparticle Solution

The synthesis of silver nanoparticle solution involves a controlled process, modified from elsewhere.^57,58^ A mixture of 14 mM ascorbic acid and 6 mM sodium citrate is stirred for 10 min, after which the pH is adjusted to 10 using ammonium hydroxide. Concurrently, silver nitrate (1.169 M) is dispersed in 8 mL of MilliQ water through sonication. The prepared silver nitrate solution is then added dropwise to the ascorbic acid-sodium citrate mixture at a rate of 1.6 mL min^–1^ under continuous magnetic stirring for 30 min, forming silver nanoparticles.

### 4.3 Preparation of GNP–Ag and SAC–Ag Solution Coating Solution

A 1:1 vol. ratio of GNP and Ag solution was added and magnetically stirred for 10 min. To produce 100 mL of GNP–Ag coating solution, 10 mL of 1:1 vol ratio of GNP–Ag mixture was added to a beaker and diluted to 100 mL using isopropanol (1:9 ratio of water: IPA). Additives include a cationic surfactant (400 ppm CTAB), PUD, and/or benzophenone that help with formulation stability,^59,60^ tackiness, and initiator, respectively.

### 4.4 Coating of Fabrics

Coating fabrics with antipathogenic formulations involves an ultrasonic atomizer mounted on a 3D-gantry system programmed to spray in a specific pattern (Figure 8). This versatile coating process can be applied to various nonwoven and medical textile materials, including spunbond, meltblown, and spun lace fabrics commonly used in medical masks and respirators.^61–63^ The treatment process begins with a fluid spray system attached to the gantry, where a syringe pump delivers the multifunctional, antipathogenic solution to a spray nozzle for aerosolization. The coating program applies two passes at a flow rate of 12 mL min^–1^, treating 40 μL cm^–2^ of the sample.

**Figure 8:**
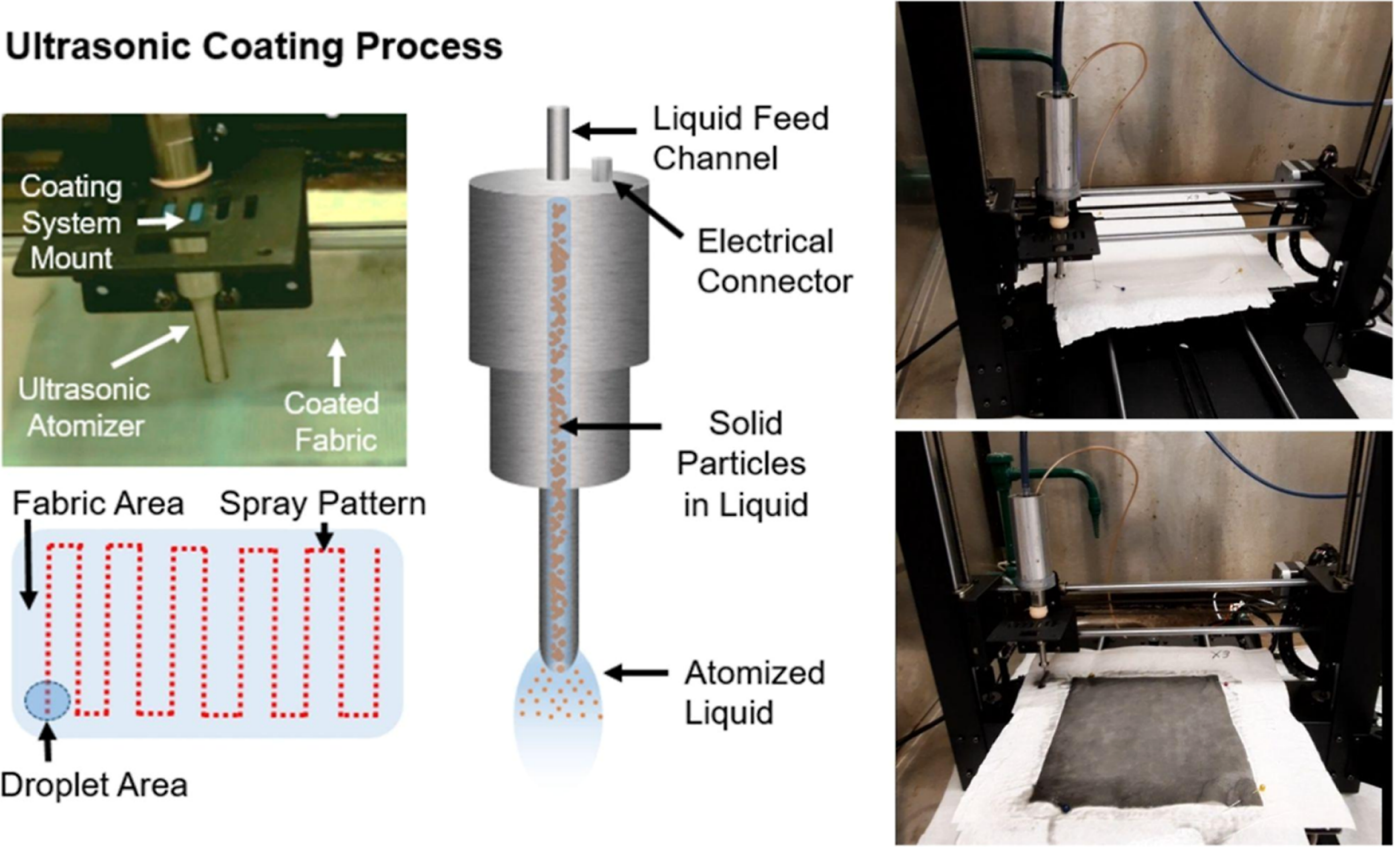
Ultrasonic coating process with depiction of GNP–Ag coating before and after ultrasonic atomization process.

After coating, the treated fabrics undergo a series of postprocessing steps. First, the coated materials are dried in a convection oven at 80 °C for 30 min. Subsequently, the samples are subjected to UV curing to cross-link PVP and PU diol polymers. This curing process is carried out using a UV LED using an Aquisense PearlBeam reactor (280 nm) or a low-pressure mercury UV lamp (254 nm, 100 mJ cm^–2^) for 2 min, sterilizing the treated fabrics.

### 4.5 Materials Characterization

#### 4.5.1 Coating Properties

The specific surface area, the density of the adsorbent, the thickness of the particulate, and the diameter of the GNP and SAC adsorbents were measured. The Ag nanoparticle size was determined using scanning electron microscopy FEI Quanta FEG 250 ESEM (with EDX) and UV–vis spectroscopy (UV-2501PC, Shimadzu). Raman spectroscopy (DXR2 Raman spectrometer, 532 nm, 10 mW laser output) was used to determine the defective (D) and graphitic (G) peaks in graphene nanoplatelets. Zeta potential was measured using the Wallis Zeta Potential Analyzer (Cordouan Technologies).

#### 4.5.2 Coated Fabric Characterization

Using an ESEM, the morphology and features of coated fabrics were characterized. EDS was used to qualitatively assess the image elements and the distribution of nanomaterials within the fabric. Raman spectroscopy (DXR2 Raman spectrometer, 532 nm, 10 mW laser output), X-ray diffraction (PANalytical X’pert Pro MRD HR-XRD), and Fourier transform infrared (FTIR) spectrometer (Bruker Tensor 27 FTIR, ATR mode, 500–4000 cm^–1^) were used to characterize the GNP–Ag-coated spunbond fabrics.

#### 4.5.3 Mechanical Testing

The fabric samples were tested using an INSTRON 5548 microtensile tester to evaluate their tensile properties. The testing followed the ASTM 5034 ^64^ and ASTM 5035 ^65^ standards, which are widely used methods for determining the breaking strength and elongation of textile fabrics. Samples measuring 2.54 cm × 5.08 cm were prepared for testing. The ASTM 5034 standard, also known as the grab test method, involves clamping the full width of the specimen in the tensile testing machine jaws, while ASTM 5035, or the strip test method, uses a narrower specimen width.

For Figure 6, statistical analysis was performed using OriginLab Pro (Version 10) to plot and analyze data. One-way ANOVA was conducted (α = 0.05) on multiple data sets. Posthoc tests were performed when a statistical significance was detected using the Tukey test method with an overall significance level of 0.05.

#### 4.5.4 Wash Testing

A wash testing protocol was implemented to assess the adhesion and stability of GNP–Ag coatings on fabric substrates. Untreated and GNP–Ag treated samples of 40 gsm M and 40 gsm SB fabrics were subjected to a washing procedure using distilled water as the wash solution. Each fabric sample was immersed in 20 mL of wash solution within a 50 mL conical tube and subjected to five 5 s vortexing cycles to simulate mechanical agitation during washing. Turbidity measurements were conducted on the resulting wash solutions using an Apera TN400 Turbidity Meter, in accordance with ISO 7027 for water quality testing.^66^ This method employs infrared light to determine sample attenuation, using a formazin solution as the reference standard. The turbidity values, expressed in Nephelometric Turbidity Units (NTU), provide a quantitative measure of suspended particles in the wash solution and indicate the degree of coating detachment from the fabric surface. Concurrently, the concentration of silver ions in the wash solution was quantified by inductively coupled plasma-mass spectrometry (ICP-MS) to assess potential leaching of Ag nanoparticles from the GNP–Ag coating.

### 4.6 Particulate Respirability Testing

Particulate respirability testing was conducted on GNP–Ag samples in accordance with ISO 18562.^67^ The testing apparatus comprises an environmental chamber with sophisticated temperature and humidity control mechanisms. A precision controller maintains the chamber conditions at 37 °C and 75% relative humidity, closely simulating the physiological parameters of human respiration. This controlled environment is crucial to ensuring that test conditions accurately reflect the challenges respiratory protective equipment faces in actual use. The chamber is further augmented with an airline connected to a laboratory source, which provides the necessary pressure to propel air through the system, mimicking the force exerted during human breathing cycles.

The airflow path in the testing setup is conditioned and exits the environmental chamber via a tube, subsequently passing through a T-junction. This junction serves a critical function in removing any condensation that may have formed, ensuring that only dry air proceeds to the subsequent stages of the testing apparatus. After the T-junction, the air stream passes through a filter, removing extraneous particles that could skew test results. After filtration, the airflow reaches the mask sample, the primary subject of the respirability test. This carefully orchestrated flow path ensures that the mask is exposed to air that closely resembles the composition and conditions of human exhalation.

The measurement and analysis of particulate matter is accomplished using a DustTrak aerosol monitor, a sophisticated instrument capable of real-time aerosol mass concentration measurements. The DustTrak employs an internal pump to draw the air sample through the testing setup, ensuring a consistent and controlled flow rate. This device measures the size-segregated mass fractions of particulates in the air, typically corresponding to PM1, PM2.5, respirable, PM10, and total PM.

### 4.7 Cytotoxicity Testing

The cytotoxicity testing protocol, adapted from ISO 18184,^68^ was designed to evaluate the efficiency of test specimens in suppressing agent activity, encompassing cytotoxicity assessment, cell sensitivity to virus reduction, and inactivation of antiviral activity. The procedure (Supporting Information—Section S2) involved plating HEL 299 cells in a 96-well plate using cell culture medium and incubating at 37 °C under 5% CO2 overnight.^69^ Test specimens, measuring 20 mm × 20 mm (∼0.1 g), were placed in 15 mL conical tubes containing 2 mL of serum-free DMEM cell culture medium. These specimens were incubated for 15 and 30 min at 33 °C. After each incubation, the tubes were vortexed for 5 s, repeated 5 times, to ensure thorough mixing of the specimen with the medium.

The resulting media was collected and diluted in 5 concentrations ranging from 10^–0^ to 10^–5^. Concurrently, the cell media in the 96-well plate were removed, and the wells were washed with fresh DMEM. The diluted media samples were then added to the prepared 96-well plate, with 100 μL of each dilution applied to six wells per condition. Control wells were filled with 2% DMEM, and blank wells were included for background subtraction. This allows for assessing potential cytotoxic effects across various concentrations and exposure times.

The plates were then incubated at 33 °C under 5% CO_2_ for an extended period, typically ten days, to allow for the development of any cytopathic effects. Throughout this incubation period, the cells were regularly monitored for signs of cytotoxicity or morphological changes. After the incubation period, cell viability was assessed using the Alamar Blue assay, an indicator of cellular metabolic activity.^70^

### 4.8 Viral Testing Using Human Coronavirus (OC43)

An end point dilution assay was used to quantify the virus required to kill 50% of infected hosts or produce a cytopathic effect in 50% of inoculated tissue culture cells (NIH/3T3 ATCC CRL-1658). The procedure is detailed in Supporting Information—Section S3. Briefly, 1.8 mL of virus suspension (2% FBS in RPMI-1640) was dispensed into a 2 mL test tube. Sample textiles (2 cm × 2 cm) were added to 20 mL conical tubes. An aliquot containing 200 μL of washout virus suspension was dispensed into the conical tube containing the sample textile and mixed for a specified contact time. An aliquot of 145 μL was transferred from the conical tube and serially diluted in a 96-well plate. The plates were incubated at 33 °C with 5% CO_2_ in an incubator for 10 days. The observed cytopathic effect in infected wells was documented, and the TCID50 was calculated using a procedure similar to ISO 18184.^68^

## Supplementary Material



## Notes

Supporting Information is available and includes additional experimental details and characterization results in the order presented in the supplementary file. This includes sedimentation and time-series imaging of GNP–Ag composite samples (Figure S1), UV treatment effects on N95 filtration media (Figure S2), detailed cytotoxicity testing procedures and plate layouts (Figure S3), and TCID_50_ antiviral testing protocols. These materials support the methodologies and findings presented in the main manuscript (PDF)
