# Supplementary material for: Ultrasonic Processing of Graphene Nanoplatelet–Silver Nanoparticle Composite Coatings for Enhanced Mechanical and Antiviral Properties in Medical Textiles
Source: ACS Omega. 2026 May 6;11(19):27762–74. doi: 10.1021/acsomega.5c06675 (PMC13191548; doi:10.1021/acsomega.5c06675)
Supplement: Supplementary file 1 [file ao5c06675_si_001.pdf]

Ultrasonic Processing of Graphene Nanoplatelet-Silver Nanoparticle Composite Coatings for Enhanced Mechanical and Anti-viral Properties in Medical Textiles

SUPPORTING INFORMATION

Robert L.F. Liang[1,2,3], Monika Snowdon[2,3], Azar Fattahi[2], Cameron Dean[2,3], Derek Eppinghoven[3], Irfani Ausri[3], Yun Wu[3], Steven Phang[2] , Aastha Gandhi[2,3], Julie Dang[2,3], Tahbit Chowdhury[2,4], Shirley Tang[3], Y. Norman Zhou[2,3], and Marina Freire Gormaly[1,*]*

[1]Department of Mechanical Engineering, York University,
4700 Keele St., Toronto, ON, Canada, M3J 1P3

[2]Centre for Advanced Materials Joining, University of Waterloo,
200 University Ave. West, Waterloo, Ontario, Canada, N2L 3G1

[3]Waterloo Institute of Nanotechnology, University of Waterloo
200 University Ave. West, Waterloo, Ontario, Canada, N2L 3G1

[4]Feedband Labs
192 Spadina Ave., Toronto, ON, M5T 2C2

Corresponding Authors:

**Robert L. F. Liang, Email: robliang@yorku.ca*

**Marina Freire-Gormaly, Email: marina.freire-gormaly@lassonde.yorku.ca*

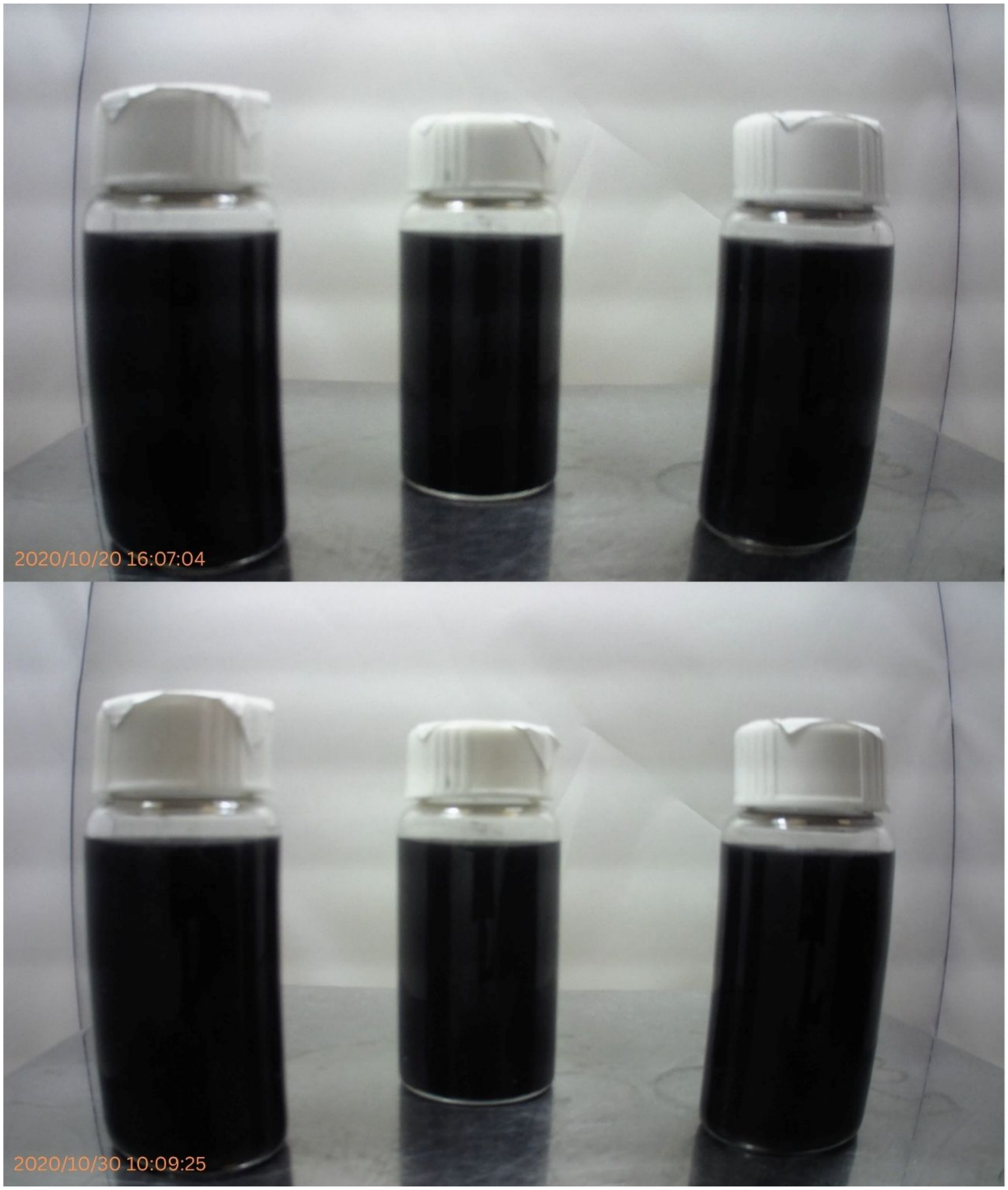

Figure S1: Camera images of GNP-Ag samples from Day 0 (top) to Day 10 (bottom)

Section 1: Effects of UV on filtration media study

Purpose: To assess the filtration efficiency of N95 filtration media after UV treatment for UV disinfection and UV curable coating applications

Test: NaCl test
Equipment: TSI 3400A aerosol generator and Scanning Mobility Particle Sizer+Electrometer (SMPS+E, Model 5.706, GRIMM Aerosol techniK, Ainring, Germany)
UV dose applied to filter: 10 000 mJ cm^{-2} (low pressure mercury, 254 nm)
Test media: 3M brand N95 filter (1” diameter)
Flow rate: 30 lpm (filtration efficiency), 0-15 lpm (pressure drop)
Results: The results indicated that the differences in filtration efficiency between UV-treated (10,000 mJ cm^{-2}) and untreated N95 masks are significant ($p < 0.0001$), however, the masks maintain a filtration efficiency above 95%. There was no significant difference in the pressure drop (airflow resistance) between UV-treated and untreated N95 masks ($p > 0.05$) in the tested flow rates between 0 lpm to 15 lpm.

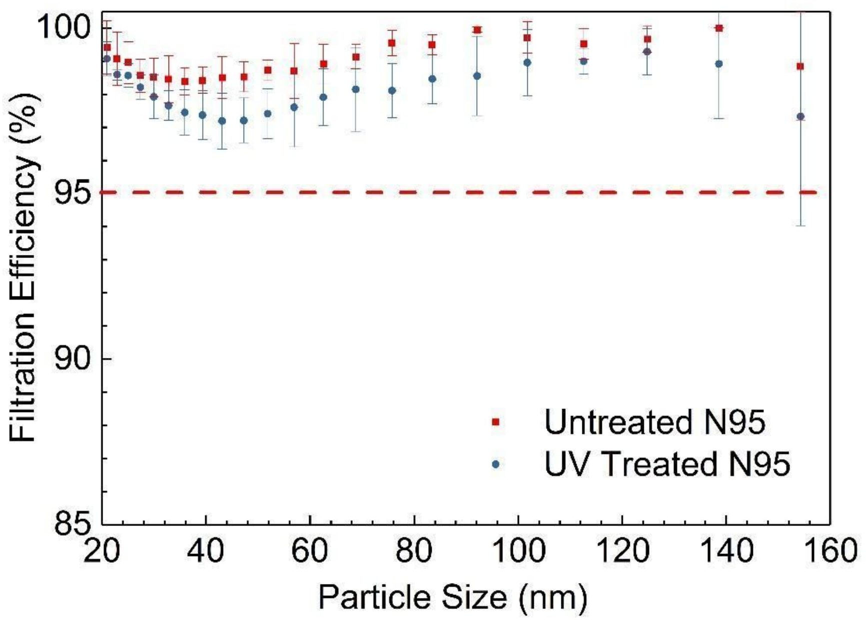

Figure S2: Filtration Efficiency of Untreated and UV treated N95 (10,000 mJ cm^{-2})

Section 2 - Cytotoxicity Study Procedure

1. Before the experiment, plate 100µL of 1.5 x 10^4 HEL 299 cells in each well of a 96-well plate, using DMEM supplemented with 10% FBS and 1% penicillin/streptomycin (P/S). Leave Row 12 (A-H) blank and add 100µL of HEL 299 cell media for background subtraction.
2. To allow cell attachment, incubate the 96-well plate at 37°C with 5% CO2 overnight (approximately 16-18 hours).
3. On Day 0, cut the test specimen into an approximately 20 mm by 20 mm piece using sterile scissors or a scalpel in a biosafety cabinet.
4. Place the specimen into a sterile 15mL falcon tube and add 2mL of serum-free DMEM.
5. Incubate the falcon tube at 33°C for 15 minutes in a water bath or incubator.
6. Before media collection, vortex the falcon tube for 5 seconds, repeating this process 5 times to ensure thorough mixing.
7. Repeat steps 4-6, but change the incubation time to 30 minutes instead of 15 minutes in step 5.
8. After collecting the media, serial dilutions are performed. Dilute the collected media into 5 different concentrations using DMEM supplemented with 2% FBS (2% DMEM), starting from 10^0 to 10^-5. Prepare at least 1 mL of each dilution.
9. Using a multichannel pipette, remove cell media from the prepared 96-well plate and wash the wells once with 100µL of serum-free DMEM.
10. Add 100µL of the undiluted media from step 8 into wells 2B-2G (6 wells per condition).
11. Repeat steps 9-10 using the five diluted media prepared in step 8, adding each dilution to a separate row of wells.
12. Add 100µL of 2% DMEM to each untreated cell well (Blue rectangle) and blank well (Red rectangle), as shown in the plate layout image (Figure S2).
13. Incubate the plate at 37°C with 5% CO2 for a predetermined period (e.g., 24, 48, or 72 hours) depending on the specific cytotoxicity assay.
14. After incubation, the chosen cell viability assay will follow the manufacturer's instructions.
15. Measure the absorbance or fluorescence using a plate reader at the appropriate wavelength for the chosen assay.
16. Calculate cell viability as a percentage of the untreated control cells and analyze the data to determine the test specimen's cytotoxic effects.

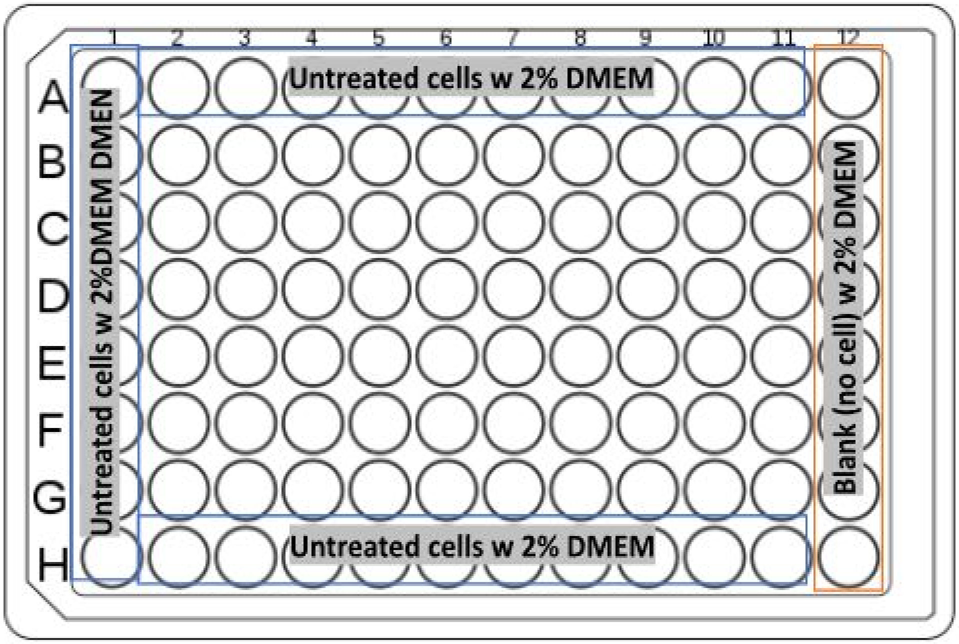

Figure S3: Plate Layout for Cytotoxicity Testing

Section 3: TCID50 Procedure

General procedure

1. Place the specimens in 50ml centrifuge tubes
2. Deposit 200 µl of the virus suspension onto the specimen at several points, ensuring even distribution.
3. Ensure the virus sample is in contact with the side containing the anti-viral material.
4. Add 10 ml of virus medium to the vial containers.
5. Vortex for 5 seconds, repeating 5 times, to remove the virus from the specimen.
6. Collect the resulting suspension for serial dilution.

Serial dilution for virus suspension (TCID50)

1. Prepare 80 ml of virus medium (2% FBS in RPMI1640) for all samples.
2. Add 1.8 ml of virus medium (2% FBS in RPMI1640) in 7 sterile 2ml test tubes
    a. 12.6ml per sample à prep 80ml
3. Add 200 µl of the diluted neutralization media from step 6 of the general procedure to the first tube and mix well—this will be the sample for row B.
    a. Perform a 1:10 serial dilution
4. Repeat procedure #2 six times for the corresponding test tubes (samples for rows B-G)
5. Transfer 140 µl of each serially diluted sample into the corresponding row (B-H) of a 96-well plate. Each well in a row should receive the same dilution.
    a. 140 µl per well x 12 wells = 1680 µL

   b. For row A, use the least diluted sample (from step 6 of the general procedure).
   c. Row H should contain only virus media as a negative control.
6. Incubate the plates in a 33C & 5% CO_2 incubator for 10 days
7. Observe cytopathic effect in infected wells and calculate the TCID50 as outlined in the ISO
